# A High Preoperative Platelet-Lymphocyte Ratio Is a Negative Predictor of Survival After Liver Resection for Hepatitis B Virus-Related Hepatocellular Carcinoma: *A Retrospective Study*

**DOI:** 10.3389/fonc.2020.576205

**Published:** 2020-10-16

**Authors:** Yun Yang, Meng-chao Wang, Tao Tian, Jian Huang, Sheng-xian Yuan, Lei Liu, Peng Zhu, Fang-ming Gu, Si-yuan Fu, Bei-ge Jiang, Fu-chen Liu, Ze-ya Pan, Wei-ping Zhou

**Affiliations:** Eastern Hepatobiliary Surgery Hospital, Shanghai, China

**Keywords:** blood platelet-lymphocyte ratio, hepatitis B virus, hepatocellular carcinoma, prognosis, CD8^+^ T-cell

## Abstract

**Objective:** To evaluate the importance of preoperative blood platelet to lymphocyte ratio (PLR) in patients with hepatitis B virus (HBV)-related hepatocellular carcinoma (HCC) after liver surgery and to examine the connection with CD8^+^ lymph cell infiltration.

**Methods:** Between 2009 and 2014, consecutive HCC patients who received curative liver surgery were included into this retrospective study. Baseline clinicopathological characteristics were analyzed to identify predictors of recurrence-free and overall patient survival rate after liver resection. The samples of all patients were under Tissue Microarray (TMA) construction and immunohistochemical staining for CD8+.The association of the number of CD8+T-cells in the cancer nests and peritumoral stroma with PLR level was analyzed.

**Results:** A total of 1,174 HBV-related HCC patients who received a liver resection without any peri-operative adjuvant therapy were enrolled into this retrospective study. Univariate and Multivariate analysis using Cox regression model showed that PLR was an independent factor affecting recurrence and overall survivals. The optimal cutoff of PLR using the receiver operating characteristic curve was 150. There were 236 patients (20.1%) who had a PLR of 150 or more. The 5-year survival rate after liver resection was 71.8% in patients with a PLR of < 150 and it was 57.2% in those with a PLR of 150 or more (*P* < 0.001). Both 5-year recurrence-free and overall survival rates in liver cancer stage A patients at Barcelona Clinic with different PLR group were also significantly different (*P* = 0.007 for recurrence and *P* = 0.001 for overall survival). Similar results were also observed in stage B patients (*P* < 0.001 for recurrence and *P* = 0.033 for overall survival). To determine the association between PLR and the severity of liver inflammation, an immuno-histological examination using CD8^+^ staining was performed on the liver specimens of 1,174 patients. Compared with low PLR (<150) group, more CD8^+^T-cells were found in the peritumoral tissue in high PLR (≥ 150) group.

**Conclusions:** PLR played as an independent factor for predicting the survival after hepatectomy for HCC patients. A high PLR was associated with an accumulation of CD8^+^ T-cells in the peritumoral stroma.

## Introduction

Inflammation has been regarded as the seventh symptom of cancer (1). Increasing evidence has shown that systemic inflammatory response (SIR) may associate with poor cancer-specific outcomes (2). The effect of SIR on carcinogenesis has been intensively studied. Current understanding suggests that SIR predisposes tumors to proliferate and metastasize through apoptosis inhibition, DNA damage, angiogenesis promotion, and tumor invasion through the upregulation of cytokines (3, 4). The presence of SIR can be determined using various markers, including c-reactive protein (CRP), absolute blood neutrophil or lymphocyte count and its ratios such as neutrophil-to-lymphocyte ratio (NLR), lymphocyte to monocyte ratio (LMR), and platelet to lymphocyte ratio (PLR). An elevation in PLR is another marker of inflammation, and has been proven to be relevant to poor clinical outcomes in different kinds of cancer patients, such as colorectal, esophageal and lung (5–7).

Recently, many researchers analyzed the impact of preoperative PLR to the survival of hepatocellular carcinoma (HCC) patients. The results of these studies are not in agreement with each other. A possible reason is that studies enrolled patients with a different pathogenesis who received discrepant treatment. Besides, a small sample size may be another reason (8–10).

In this paper, we assessed the significance of preoperative PLR in a large cohort of HCC patients with HBV infection who had undergone a potentially curative surgery and to examine the connection with CD8^+^ lymph cell infiltration.

## Patients and Methods

### Patients

This study enrolled patients with HBV-related HCC who received a liver resection carried out by one surgical team at the Eastern Hepatobiliary Surgery Hospital in Shanghai between January 2009 and June 2014. The diagnosis of HCC before surgery was relied on the diagnostic criteria for HCC according to the European Association for the Study of the Liver (EASL) (11). The pre-operative diagnosis of HCC was verified after the surgery according to the pathologic examinations. The inclusion criteria for this research were pre-operative World Health Organization (WHO) performance status of 0-1; Child-Pugh class A; no macrovascular invasion; no distant metastasis; no chemotherapy, radiotherapy, radiofrequency ablation, or percutaneous ethanol injection before liver resection; curative resection; and resected specimens confirmed as HCC on pathological result.

liver transplantation (LT) is the best treatment option for HCC patients with liver cirrhosis because it eliminates the tumor and the underlying cirrhotic tissue simultaneously. However, LT is not offered to all cirrhotic patients with HCC. In our hospital, patients within Milan criteria would be recommended to receive LT. The reasons why the patients who met the Milan criteria did not receive LT in this study were: (1) refusal to LT; (2) failure of affording the high cost of LT; (3) organ shortage; (4) concern about severe adverse effects of long term oral immunosuppressive agents after LT. In addition, previous studies indicated that overall survival of patients with Milan criteria after liver resection (LR) were comparable to those after LT (12–14). This observation may be attributable to advances in liver surgery, perioperative therapies for patients with liver cirrhosis, and the development of advanced multimodality for the recurrent lesions. Therefore, in this study, for HCC patients with cirrhosis, LR was performed instead of LT.

The definition of curative tumor resection was the complete macroscopic and microscopic removal of all tumors. The maximal diameter of liver tumor was regarded as the tumor size. The patterns of liver surgery were designed based on the lesion size, location, as wells as the residual liver volume. Remove of fewer than three couinaud liver segments was defined as minor liver resection, while remove of three or more liver segments was defined as major liver resection. The clinical staging was determined by the Barcelona Clinic Liver Cancer (BCLC) staging system (11). The histological grade of cirrhosis and inflammation in peritumor tissue was evaluated according to the Ishak classification (15). The histological grade of tumor differentiation was determined by reference to the Edmondson Steiner grading system.

All HBV-related HCC patients had a chest X-ray, ultrasonography (USG), contrast computed tomography (CT) or magnetic resonance imaging (MRI) of their liver. Laboratory blood tests were used to obtain the following: hepatitis B surface antigens (HBsAg) and HBeAg, hepatitis C virus antibody (HCV-Ab), serum alpha-Fetoprotein (AFP), carcinoembryonic antigen (CEA) and carbohydrate antigen 19-9 (CA19-9) antigens, white blood cell/neutrophil/lymphocyte/monocyte/platelet (PLT) counts, serum albumin, serum total bilirubin, alanine (ALT) and aspartate (AST) aminotransferases, and prothrombin time (PT). All patients were positive for HBsAg and negative for HCV-Ab. Written informed consent was obtained from all patients. The study was approved by the Institutional Review Board of the Eastern Hepatobiliary Surgery Institute, Shanghai, China.

### Follow-Up and Treatment for Tumor Recurrences

The patients were reviewed once every 3 months within the beginning 2 years, and then once every 6 months afterwards. All review procedure was conducted by hospital staff who were blinded from this research. All patients were reviewed for post-operative recurrence with regular assessment using AFP, chest x-ray and abdominal USG. A CT and (or) MRI examination were performed every 3 months after lever resection. The diagnostic criterion for HCC recurrence were the same as for pre-operative diagnosis. If recurrence was confirmed, the tumors were treated aggressively through a multimodal treatment that included re-resection, transarterial chemoembolization (TACE), percutaneous radiofrequence ablation (PRFA), and percutaneous ethanol injection (PEI). The procedure was decided by the tumor recurrence pattern, reserved liver function and general condition of the patient when the recurrence was diagnosed.

During the study period, a total of 723 patients suffered from tumor recurrence. Of 723 patients, there were 602 patients developed intrahepatic recurrence, 59 patients with extrahepatic metastasis, and 62 patients with synchronous intrahepatic and extrahepatic recurrences. Because extrahepatic metastasis is a contraindication of liver transplantation, 121 patients with extrahepatic metastasis were not recommended for liver transplantation. As described above, in our hospital, Milan criteria was used to select patients for liver transplantation. Of 602 patients with intrahepatic recurrence, only 276 patients with tumor recurrence met Milan criteria. And liver transplantation was recommended. However, they did not receive liver transplantation. The reasons why these 276 patients did not receive liver transplantation as the first treatment were due to organ shortage (*n* = 73), refusal to liver transplantation (*n* = 105) or socio-financial reasons (*n* = 98).

### Tissue Microarray (TMA) and Immunohistochemical Staining

The samples of all patients were under TMA construction and immunohistochemical staining analysis as previously described (16). The primary antibodies used were mouse monoclonal anti-CD8 (1:100, Abcam). Microarrays were assessed through two-hundred times magnification under light microscopy by 2 independent observers who had no knowledge of the patient's clinicopathologic data. Any discrepancies were quickly resolved by discussion between the observers. For CD8 staining, positive cells in each 1-mm-diameter cylinder were counted and expressed as the mean value of the triplicates (cells/spot).

### Propensity Score Matching (PSM)

To reduce potential biases which are inherent in retrospective studies, propensity score matching (PSM) was used. Patients with high PLR (≥150) were matched with patients with low PLR (<150) using the PSM as previous description (17, 18). Covariates entered into the PSM model included hepatitis B e antigen (HbeAg), alpha-fetoprotein (AFP), aspartate aminotransferase (AST), HBV-DNA load, Ishak inflammation score, tumor diameter, tumor encapsulation, microvascular invasion, tumor number, extent of liver resection, and tumor differentiation. PSM was performed as a 1:1 matching between patients with high PLR (≥150) and the low PLR (<150). The matching procedure has been described previously (19).

### Statistical Analysis

Recurrence-free survival (RFS) and overall survival were used as primary endpoints. RFS was counted from the date of the operation to the date of detection of recurrence, or censored at the last known review date. Overall survival (OS) was defined as the interval between the date of operation and death. Patients clinical and follow-up data were collected from the clinical records and the hospital cancer data center. This study was censored on May 31, 2019. For individuals who followed-up before that date, a censor was applied on the last date the patient was evaluated either radiologically or clinically, and was found to have no recurrence for RFS, and on the last date the patient was known to be alive for OS.

All data were shown as the mean ± standard deviation (SD) or median (range). The characteristics of HCC cases were compared using a student two-sample unpaired *t*-test for continuous variables and a χ^2^ test for categorical variables. Kaplan-Meier method was used to analyze RFS and OS curves. Differences between RFS and OS curves were evaluated by the log-rank test. Cox proportional hazard regression models were used to identify the independent factors of recurrence and overall survival based on the variables selected in the univariate analysis. Clinicopathological characteristics that might be associated with the number of CD8^+^T-cells in the peritumoral stroma were evaluated by univariate logistic regression analysis, and the variables that were significant (*P* < 0.05) were subjected in the stepwise multivariate analysis. Statistical analysis was performed with SPSS 13.0 (SPSS Inc, Chicago IL).

We conducted nomograms on the results of multivariate analysis in the entire cohort and by the package of rms in R version 3.5.1 (http://www.r-project.org/). The accuracy of prediction of nomogram was quantified by the concordance index (C-index). The difference of C-index between nomograms and other predictors were compared by the rcorrp.cens in Hmisc in R (20) The prediction of survival between nomogram and other predictors was compared using ROC curve analysis. Definition of statistical significance was *P* < 0.05 in two-tailed.

## Results

### Baseline Characteristics

During the investigation period, 1,211 patients with HBV-related HCC received a liver resection with curative intent, and they were enrolled into this study. Thirty seven patients were excluded from this study because of an early metastasis and (or) HCC recurrence within 1 month of surgery (*n* = 11), preoperative hepatic arterial chemoembolization (*n* = 5), death within 30 days of surgery due to liver failure (*n* = 6), or clinical evidence of infection or other inflammatory conditions before surgery (*n* = 15). After exclusion, 1,174 HCC patients remained for analysis (Supplementary Figure 1).

The background clinical characteristics of the cases are listed in Supplementary Table 1. The median age was 50 (range 20–70) years. 1035 (88.16%) patients were males and 139 (11.84%) females. All patients were positive for HBsAg and negative for anti-HCV. 281 (23.93%) patients were HBeAg positive, and the remaining 893 (76.07%) patients were negative for HBeAg. All patients are with a preserved liver function of Child-Pugh A grade. The median inflammation score of the patients was 6 (range 2–14), and the median fibrosis score of the patients was 4 (range 1–6). There were 552 (47.02%) patients with their HBV-DNA level ≥2,000 IU/ml. The diameters of the initial tumors ranged from 0.5 to 22 cm (median 4.9 cm). Using the BCLC staging, 695 patients (59.20%) were in BCLC stage 0 and A, 479 patients (40.80%) were in stage B. Of the 1,174 patients, there were 425 patients (36.20%) who had microvascular invasion. There were 521 (44.38%) patients who had multiple tumor nodules, and 596 (50.77%) patients who had complete tumor encapsulation. There were 230 patients received major liver resection. The tumors were well-differentiated in 245 patients (20.87%) (E-S grades I and II), and poorly differentiated in 929 patients (79.13%) (E-S grades III and IV).

The censor date of the investigation was May 31, 2019. The median follow-up time was 40.2 months (range 3.3 – 125.0 months).

### Association of PLR With Clinico-Pathological Characteristics

Receiver operating characteristic (ROC) curve analysis was used to determine the cutoff value for PLR. An optimal cutoff value for PLR was 150, and this cutoff value was used to categorize between the high and the low PLR groups. The area under curve is 0.743, the specificity is 0.738, and the sensitivity is 0.710 (Supplementary Figure 2). 236 (20.10%) patients were categorized into the high (≥150) PLR group, and 938 (79.90%) patients were categorized into the low (<150) PLR group. Table 1 shows the relationships of the clinico-pathological characteristics between the two groups of patients. The high PLR group had a significantly higher AFP level, higher aspartate aminotransferase value, higher Ishak inflammation score, higher viral load (≥2,000 IU/ml), larger tumor size, poorer tumor differentiation, and larger proportion of HBeAg positive patients (all *P* < 0.05), although no significant differences were found in age, gender, and Ishak fibrosis score (all *P* > 0.05). More patients received major liver resection in the high PLR group than the low PLR group (*P* < 0.001). Besides, the high PLR group had significantly more patients with multiple tumor nodules, no tumor encapsulation, and more patients with microvascular invasion than the low PLR group (all *P* < 0.05). Thus, a high PLR was associated with advanced malignant characteristics of HCC. Platelet, white blood cell and lymphocyte counts was also listed in Table 1, the low PLR group had a significantly lower platelet counts, and higher lymphocyte counts (all *P* < 0.001), however, the difference in white blood cell counts between two groups was not significant (*P* > 0.05).

**Table 1:** Comparison of Clinicopathological and Demographic Characteristics of Patients With Elevated and Low PLR.

	**Low PLR (<150), *N* = 938**	**Elevated PLR (≥150), *N* = 236**	***P*-value**
**Gender**			
Male	831 (88.59)	204 (86.44)	0.361
Female	107 (11.41)	32 (13.56)	
Age (years)^a^	49.99 ± 10.28	50.06 ± 11.06	0.166
Liver Cirrhosis			0.486
Yes	565 (60.23)	148 (62.71)	
No	373 (39.77)	88 (37.29)	
HBeAg			0.013
Positive	210 (22.38)	71 (30.08)	
Negative	728 (77.62)	165 (65.92)	
AFP (ng/ml)			0.020
≥20	567 (60.45)	162 (68.64)	
<20	371 (39.55)	74 (31.36)	
Alanine aminotransferase (U/L)			0.152
≥40	440 (46.91)	123 (52.12)	
<40	498 (53.09)	113 (47.88)	
Aspartate aminotransferase (U/L)			<0.001
≥40	384 (40.94)	137 (58.05)	
<40	554 (59.06)	99 (41.95)	
Total bilirubin (ummol/ml)			0.646
≥17.1	341 (36.35)	82 (34.75)	
<17.1	597 (63.65)	154 (65.25)	
Albumin (g/L)			0.476
≥35	893 (95.20)	222 (94.07)	
<35	45 (4.80)	14 (5.93)	
HBV DNA (IU/ml)			0.041
≥2,000	427 (45.52)	125 (52.97)	
<2,000	511 (54.48)	111 (47.03)	
Ishak inflammation score^a^	5.12 ± 1.73	6.34 ± 2.98	0.002
Ishak fibrosis score^a^	4.73 ± 1.56	5.08 ± 1.36	0.251
Tumor diameter (cm)^a^	5.47 ± 3.67	8.64 ± 4.34	<0.001
Tumor encapsulation			<0.001
None	504 (53.73)	145 (61.02)	
Complete	434 (46.27)	91 (38.98)	
**Major resection**			
Yes	154 (16.41)	76 (32.20)	<0.001
No	784 (83.59)	160 (67.80)	
Microvascular invasion			0.012
Yes	323 (34.43)	102 (43.22)	
No	615 (65.57)	134 (56.78)	
Tumor number			<0.001
Single	553 (58.96)	100 (42.37)	
Multiple	385 (41.04)	136 (57.63)	
Tumor differentiation			0.001
I/II	214 (22.81)	31 (13.14)	
III/IV	724 (77.19)	205 (86.86)	
Platelet Counts (*10^9^/L)	134.10 ± 56.31	231.63 ± 82.37	<0.001
White blood cell Counts (*10^9^/L)	4.61 ± 1.80	4.78 ± 1.28	0.121
Lymphocyte Counts (*10^9^/L)	1.61 ± 0.63	1.12 ± 0.38	<0.001

a *Age, Ishak inflammation score, Ishak fibrosis score, and tumor diameter are expressed as mean ± SD*.

*HBeAg, hepatitis B e antigen; AFP, alpha-fetoprotein*.

PSM analysis created 226 pairs of patients. Baseline characteristics of the patients in the propensity matched cohort are listed in Supplementary Table 2. After PSM, no significant differences were found in all clinicopathological variables between the two groups (*P* > 0.05).

### Correlation of PLR With Prognosis of Patients With HCC

**Figure 1:**
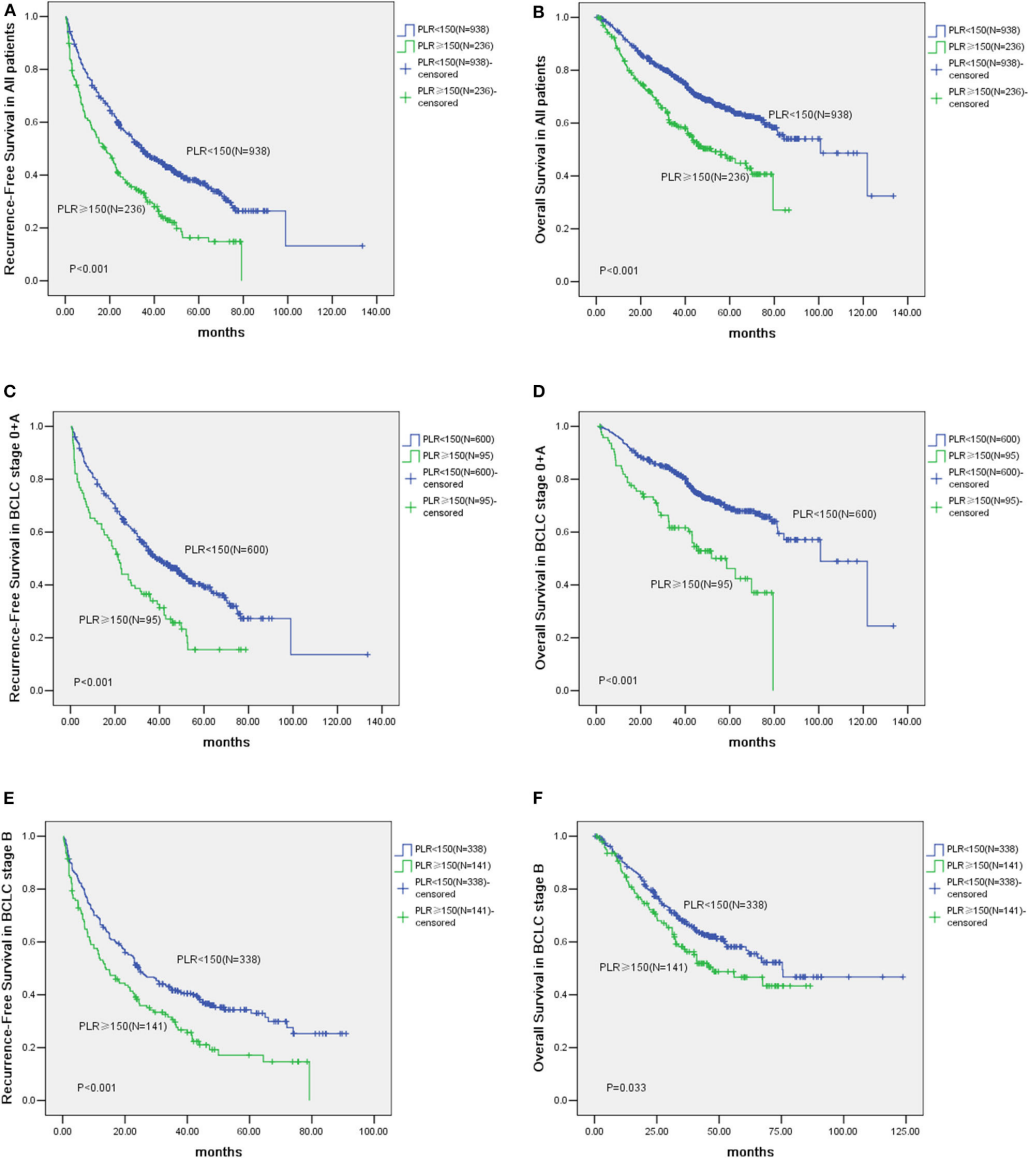
Kaplan-Meier analysis of 1,174 Hepatocellular Carcinoma (HCC) patients and survival curves of 695 Barcelona Clinic Liver Cancer (BCLC) stage 0 + A patients and survival analysis of HCC patients in BCLC stage B. **(A)** The cumulative Recurrence-Free Survival (RFS) curve of HCC patients with high PLR (≥150) and patients with low PLR (<150) (*P* < 0.001). **(B)** The cumulative Overall Survival (OS) curve of HCC patients with high PLR (≥150) and patients with low PLR (<150) (*P* < 0.001). **(C)** The cumulative RFS curve of patients with high PLR (≥150) and patients with low PLR (<150) in BCLC stage 0 + A (*P* < 0.001). **(D)** The cumulative OS curve of patients with high PLR (≥150) and patients with low PLR (<150) in BCLC stage 0 + A (*P* < 0.001). **(E)** The cumulative Recurrence-Free Survival (RFS) curve of patients with elevated PLR (≥150) and other patients with low PLR (<150) in BCLC stage B (*P* < 0.001). **(F)** The cumulative Overall Survival (OS) curve of patients with elevated PLR (≥150) and other patients with low PLR (<150) in BCLC stage B (*P* = 0.033).

The median follow-up of all the HCC cases was 40.2 months. The Kaplan-Meier survival curves comparing the low vs. the high PLR groups are shown in Figures 1A,B. The cumulative 1, 3 and 5-year recurrence-fee survival (RFS) rates of the high PLR group (≥150) were significantly lower than the low PLR group (57.30, 30.92, and 16.35%, respectively, vs. 75.71, 48.13, and 36.92%, respectively, log-rank test, *P* < 0.001). Likewise, high PLR (≥150) was negatively correlated with the cumulative 1, 3, and 5-year overall survival (OS) rates of 83.92, 59.13, and 44.92%, respectively, for the high PLR group vs. 92.51, 77.06, and 64.51%, respectively, for the low PLR group, log-rank test (*P* < 0.001).

According to the BCLC classification, 695 patients were classified into stage 0 and A. Of these patients, 95 patients are with their PLR ≥150 and 600 have low PLR (<150). The prognosis of patients with elevated PLR (≥150) are worse than those with low PLR (<150). For example, the 1, 3, and 5-year RFS rates were 63.25, 35.37, and 19.53% vs. 80.09, 51.73, and 39.21%, respectively (*P* < 0.001). Whereas, the 1, 3, and 5-year OS rates were 81.92, 61.63, and 42.45% vs. 94.73, 82.15, and 68.65%, respectively (*P* < 0.001) (Figures 1C,D).

Of patients in the BCLC stage B, the prognosis of cases (*n* = 141) with high lPLR (≥150) was poorer than those patients (*n* = 338) with low levels of PLR (<150). For example, the 1, 3, and 5-year RFS rates were 53.21, 29.73, and 17.11% vs. 66.51, 41.76, and 33.05%, respectively (*P* < 0.001). Whereas, the 1, 3, and 5-year OS rates were 85.71, 57.32, and 43.31% vs. 89.72, 67.65, and 56.82%, respectively (*P* = 0.033) (Figures 1E,F).

After PSM, the 1, 3, and 5 year RFS rates of high PLR (≥150) group were 58.52, 33.53, and 16.95%, respectively, while the corresponding figures for the patients with low PLR (<150) were 68.91, 43.32, and 34.44%, respectively. The cumulative RFS of high PLR (≥150) group was significantly lower than that of the low PLR (<150) group after PSM (*P* = 0.001). After PSM, the 1, 3, and 5 year cumulative OS rates of the patient with high PLR (≥150) were 83.81, 59.33, and 46.32%, respectively, compared with the patients with low PLR (<150) of 87.52, 66.83, and 55.37%, respectively. Thus, the OS rates of cases with high PLR (≥150) were significantly lower than those in cases with low PLR (<150) in the PSM cohort (*P* = 0.032) (Supplementary Figures 3A,B).

### Factors Affecting Prognosis of HCC in the Total Study Population

Independent risk factors for RFS and OS were identified using cox regression analyses. Univariate regression analysis indicated that alanine and aspartate aminotransferase levels, HBV DNA level, Ishak inflammation score, PLR, AFP, tumor encapsulation, microvascular invasion, multiple HCC, tumor differentiation, tumor size, and cirrhosis were each associated significantly with worse RFS (Table 2). Whereas, alanine and aspartate aminotransferase levels, albumin, HBV DNA level, PLR, AFP, tumor encapsulation, microvascular invasion, multiple HCC, tumor differentiation, and tumor size were associated with worse OS (Table 3). Multivariate Cox regression analysis indicated HBV DNA level ≥2,000 IU/ml (HR = 1.235; *P* = 0.013), Ishak inflammation score ≥3 (HR = 1.116; *P* = 0.035), PLR ≥150 (HR = 1.494; *P* < 0.001), AFP ≥20 ng/ml (HR = 1.363; *P* < 0.001), absence of complete tumor encapsulation (HR = 0.785; *P* = 0.006), microvascular invasion (HR = 1.126; *P* = 0.017), multiple HCC (HR = 1.216; *P* =0.015), and tumor size ≥5 cm (HR = 1.285; *P* = 0.003) were independent risk predictors of worse RFS (Table 2), whereas aspartate aminotransferase ≥40 U/L (HR = 1.531; *P* = 0.001), HBV DNA level ≥2,000 IU/ml (HR = 1.485; *P* = 0.001), PLR ≥150 (HR = 1.327; *P* = 0.017), AFP ≥20 ng/ml (HR = 1.424; *P* = 0.004), absence of complete tumor encapsulation (HR = 0.780; *P* = 0.029), and tumor size ≥5 cm (HR = 1.692; *P* < 0.001) were independent risk predictors of worse OS (Table 3).

**Table 2:** Univariate and Multivariate analysis of factors associated with Recurrence-free survival of patients with HCC.

	**Hazard ratio (95%CI)**	***P*-value**
**Univariate analysis**		
Gender (male vs. female)	0.901 (0.719–1.129)	0.367
Age (year) (≤60 vs. >60)	0.848 (0.695–1.035)	0.105
Alanine aminotransferase (≥40 vs. <40 U/L)	1.212 (1.047–1.302)	0.020
Aspartate aminotransferase (≥40 vs. <40 U/L)	1.504 (1.300–1.741)	<0.001
Albumin (<35 vs. ≥35 g/L)	0.806 (0.588–1.105)	0.180
HBV DNA (≥2,000 vs. <2,000 IU/ml)	1.577 (1.564–1.883)	<0.001
Ishak inflammation score (≥3 vs. <3)	1.227 (1.156–1.345)	0.014
Ishak fibrosis score (≥3 vs. <3)	0.825 (0.731–1.267)	0.328
PLR (≥150 vs. <150)	1.747 (1.474–2.069)	<0.001
AFP (≥20 vs. <20 ng/ml)	1.649 (1.409–1.929)	<0.001
HBeAg (positive vs. negative)	1.166 (0.995–1.366)	0.058
Tumor encapsulation (yes vs. no)	0.698 (0.603–0.809)	<0.001
Major resection (yes vs. no)	1.168 (0.973–1.403)	0.096
Microvascular invasion (yes vs. no)	1.575 (1.343–1.847)	<0.001
Tumor number (multiple vs. single)	1.679 (1.377–2.048)	<0.001
Tumor differentiation (III+IV vs. I+II)	1.560 (1.281–1.899)	<0.001
Tumor diameter (≥5 vs. <5 cm)	1.644 (1.419–1.904)	<0.001
Liver cirrhosis (yes vs. no)	1.256 (1.176–1.442)	0.003
**Multivariate analysis**		
HBV DNA (≥2,000 vs. <2,000 IU/ml)	1.235 (1.133–1.465)	0.013
Ishak inflammation score (≥3 vs. <3)	1.116 (1.016–1.278)	0.035
PLR (≥150 vs. <150)	1.494 (1.350–1.786)	<0.001
AFP (≥20 vs. <20 ng/ml)	1.363 (1.254–1.610)	<0.001
Tumor encapsulation (yes vs. no)	0.785 (0.712–0.879)	0.006
Microvascular invasion (yes vs. no)	1.126 (1.114–1.357)	0.017
Tumor number (multiple vs. single)	1.216 (1.128–1.424)	0.015
Tumor diameter (≥5 vs. <5 cm)	1.285 (1.188–1.518)	0.003

*HRs(95% CI) and P-values were calculated using univariate and multivariate Cox proportional hazard regression*.

*HBeAg, hepatitis B e antigen; AFP, alpha-fetoprotein*.

**Table 3:** Univariate and Multivariate analysis of factors associated with Overall survival of patients with HCC.

	**Hazard ratio (95%CI)**	***P*-value**
**Univariate analysis**		
Gender (male vs. female)	0.918 (0.674–1.249)	0.585
Age (year) (≤60 vs. >60)	0.983 (0.754–1.280)	0.897
Alanine aminotransferase (≥40 vs. <40 U/L)	1.334 (1.091–1.631)	0.005
Aspartate aminotransferase (≥40 vs. <40 U/L)	2.040 (1.664–2.501)	<0.001
Albumin (≥35 vs. <35 g/L)	0.636 (0.433–0.934)	0.021
HBV DNA (≥2,000 vs. <2,000 IU/ml)	1.631 (1.292–2.058)	<0.001
Ishak inflammation score (≥3 vs. <3)	1.127 (0.786–1.635)	0.074
Ishak fibrosis score (≥3 vs. <3)	1.072 (0.821–1.782)	0.102
PLR (≥150 vs. <150)	1.891 (1.515–2.361)	<0.001
AFP (≥20 vs. <20 ng/ml)	2.073 (1.648–2.608)	<0.001
HBeAg (positive vs. negative)	1.160 (0.934–1.441)	0.180
Tumor encapsulation (yes vs. no)	0.507 (0.412–0.624)	<0.001
Major resection (yes vs. no)	1.094 (0.852–1.406)	0.481
Microvascular invasion (yes vs. no)	2.066 (1.680–2.539)	<0.001
Tumor number (multiple vs. single)	2.162 (1.680–2.781)	<0.001
Tumor differentiation (III+IV vs. I+II)	2.433 (1.758–3.366)	<0.001
Tumor diameter (≥5 vs. <5 cm)	2.628 (2.124–3.251)	<0.001
Liver cirrhosis (yes vs. no)	1.194 (0.976–1.460)	0.084
**Multivariate analysis**		
Aspartate aminotransferase (≥40 vs. <40 U/L)	1.531 (1.180–1.985)	0.001
HBV DNA (≥2,000 vs. <2,000 IU/ml)	1.485 (1.160–1.901)	0.001
PLR (≥150 vs. <150)	1.327 (1.053–1.674)	0.017
AFP (≥20 vs. <20 ng/ml)	1.424 (1.120–1.810)	0.004
Tumor encapsulation (yes vs. no)	0.780 (0.624–0.974)	0.029
Tumor diameter (≥5 vs. <5 cm)	1.692 (1.340–2.136)	<0.001

*HRs (95% CI) and P-values were calculated using univariate and multivariate Cox proportional hazard regression*.

*HBeAg, hepatitis B e antigen; AFP, alpha-fetoprotein*.

Additional cox regression analyses was performed to assess the Independent risk factors for RFS and OS in the PSM cohort. Univariate and multivariate analyses of RFS and OS after liver surgery in the PSM cohort are indicated in Supplementary Tables 3, 4. On multivariate analyses, high PLR (≥150) remained independently associated with a worse RFS (HR 1.487, 95% CI 1.182–1.870; *P* = 0.001) and OS (HR 1.309, 95% CI 1.201–1.738; *P* = 0.041).

### Construction Nomograms and Comparison of the Performance Between Nomograms and Predictors

**Figure 2:**
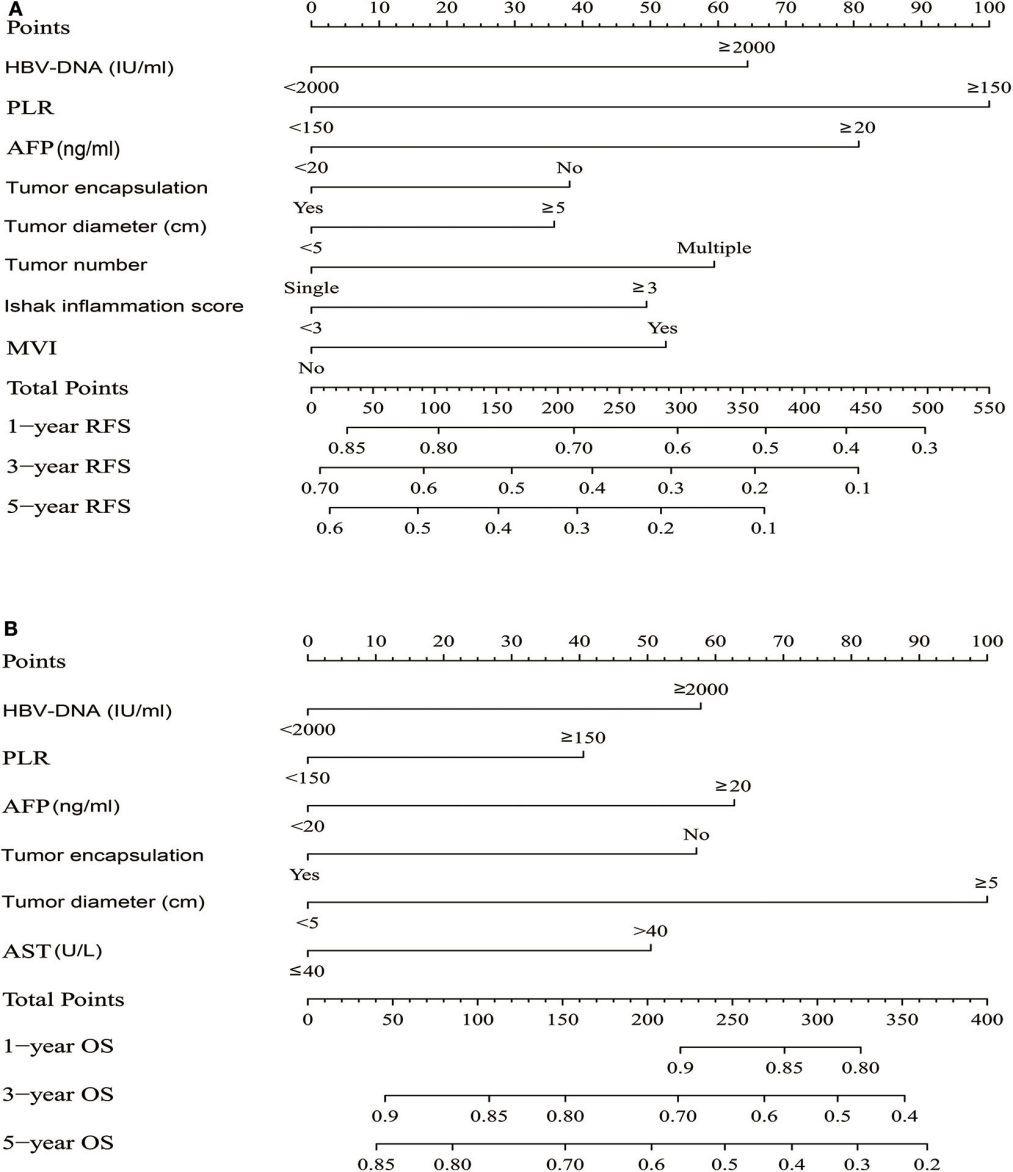
HCC patients survival nomogram. **(A)** HCC patients survival nomogram for Recurrence-Free Survival. **(B)** HCC patients survival nomogram for Overall Survival. (To use the nomogram, individual patient's value is located on each variable axis, and a line is drawn upward to determine the number of points received for each variable value. The sum of these numbers is located on the Total Points axis, and a line is drawn downward to the survival axes to determine the likelihood of 1-, 3-, or 5-year survival).

The independent risk factors derived from multivariate analysis of RFS in all patients were performed to construct the RFS nomogram (Figure 2A). Similarly, the OS nomogram analysis were also performed (Figure 2B). The C-index of nomograms for RFS and OS were 0.649 (95% CI: 0.626–0.671) and 0.716 (95% CI: 0.685–0.746), respectively. Other predictors including the independent risk factors were compared with the nomograms to determine the accuracy of prediction from different models (Supplementary Table 5). The C-index of the nomogram for RFS was 0.649, which was statistically higher than the PLR (0.549), AFP (0.564), tumor encapsulation (0.561), tumor diameter (0.582), HBV-DNA (0.542), tumor number (0.538), ishak inflammation score (0.567) and MVI (0.559) (all *P* < 0.001). As for the OS, the C-index of the nomogram was 0.716, which was statistically higher than the PLR (0.559), aspartate aminotransferase (0.591), AFP (0.584), tumor encapsulation (0.591), HBV-DNA (0.569) and tumor diameter (0.635) (all *P* < 0.001). These data illustrated a more predication accuracy of the established nomograms than other predictors. ROC curve analyses showed the nomograms for RFS and OS both had a larger AUC than any other independent risk factors (Supplementary Table 6).

### Prognostic Value of PLR for HCC Patients Survival

With respect to RFS, the C-index of PLR was 0.549, and it was significantly higher than tumor number (*P* = 0.005) (Supplementary Table 5). In the ROC curve analysis for RFS (Supplementary Table 6), there was no significantly difference between PLR and other predictors, except for tumor number. PLR had a larger AUC than tumor number (*P* = 0.048). In the multivariate analysis of RFS, the HR for PLR was the highest. We also observed that PLR had the highest specific weight in the nomogram for RFS (Figure 2A). Therefore, we demonstrated that PLR was the best predictor of recurrence. As for OS, The C-index of PLR was 0.559, which was the lowest among the predictors (Supplementary Table 5). In the ROC curve analysis for OS (Supplementary Table 6), PLR also had the lowest AUC value than other predictors. We also observed that PLR had the lowest specific weight in the nomogram for OS (Figure 2B). Thus, we combined the PLR with other predicters to identify the best predictors for OS. In the Supplementary Table 6, PLR combined with tumor diameter had a larger AUC than other combinations. Thus, PLR combined with tumor diameter may be the best predictor for OS.

### Immunohistochemical Analysis

**Figure 3:**
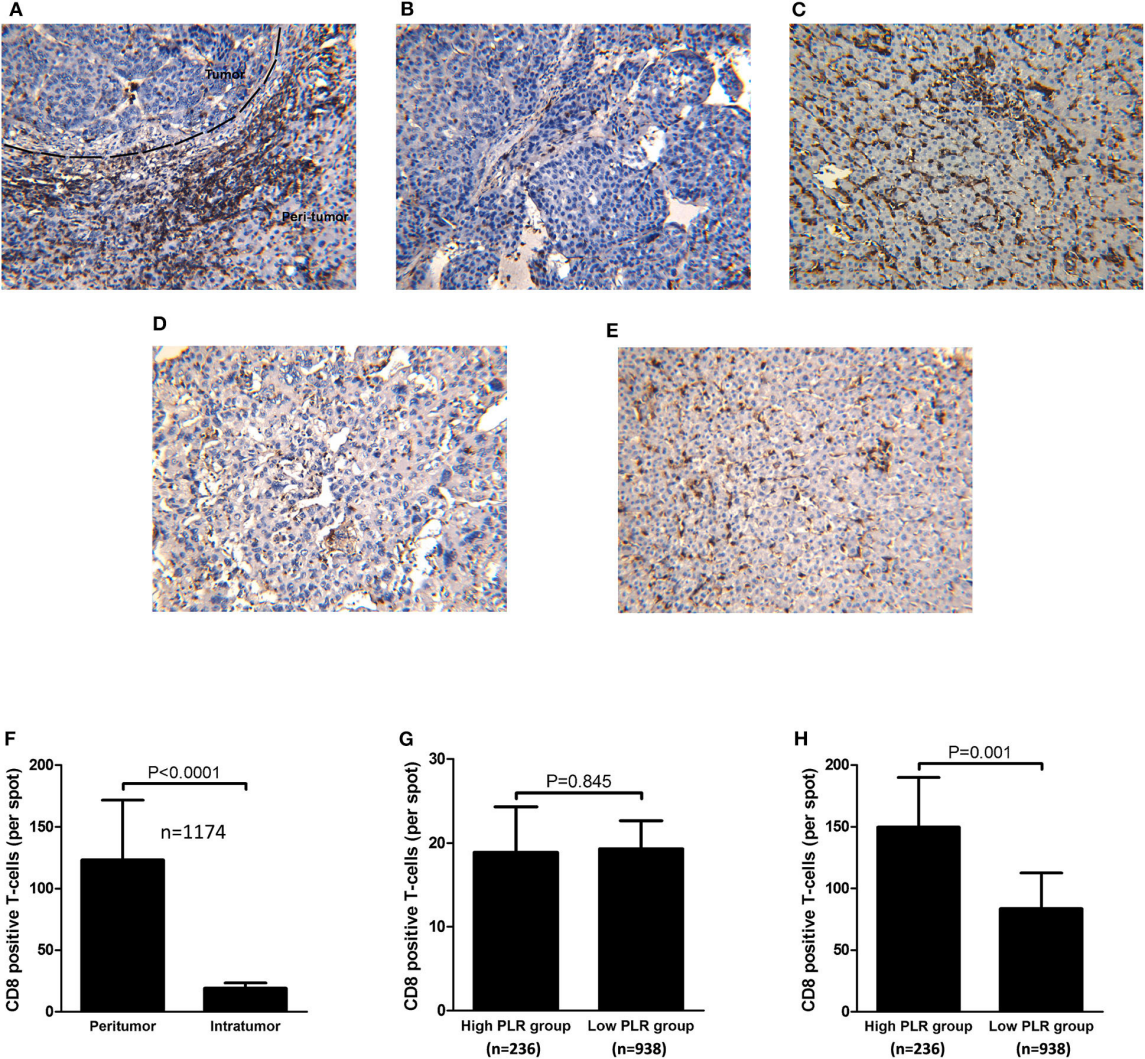
Immunohistochemical staining of intratumoral and peritumoral CD8 from consecutive tissue microarrays and distribution of CD8^+^ T-cells and correlations with PLR level. **(A)** Immunohistochemical staining of CD8 throughout the tissue. **(B)** Immunohistochemical staining of CD8 in HCC tissues of patients with high PLR (≥ 150). **(C)** Immunohistochemical staining of CD8 in tumorside tissues of patients with high PLR (≥150). **(D)** Immunohistochemical staining of CD8 in HCC tissues of patients with low PLR (<150). **(E)** Immunohistochemical staining of CD8 in tumorside tissues of patients with low PLR (<150). **(F)** CD8^+^ T-cells were more abundant in peritumoral tissue than in tumor tissue. **(G)** There was no significant difference of intratumoral CD8^+^ T-cell counts between patients with high PLR (≥150) and low PLR (<150). **(H)** Peritumoral CD8^+^ T-cells were more abundant in HCC patients with high PLR (≥150) than patients with low PLR (<150).

All of the samples of 1,174 patients were under TMA construction and immunohistochemical staining for CD8^+^T-cells. As shown in Figure 3A, CD8^+^ T-cells were present throughout the tissue samples, but they were often predominant in the peritumoral stroma rather than in the cancer nests (123.2 ± 48.6 and 19.1 ± 4.4 cells/field, respectively; *P* < 0.001; Figures 3A,F). When tumor infiltration by CD8^+^ T-cells between the high and low PLR groups were compared, there was no significant difference in intratumoral CD8^+^ T-cell counts between the two groups (18.9 ± 5.4 vs. 19.3 ± 3.3 *P* = 0.845; Figures 3B,D,G). However, peritumoral stroma in the high PLR group has significantly more CD8^+^T-cells than that in the low PLR group (149.7 ± 40.3 vs. 83.5 ± 29.0 *P* = 0.001; Figures 3C,E,H).

### Association of CD8^+^T-Cells in the Peritumoral Stroma With Clinico-Pathological Characteristics

There was no significant difference between intra tumoral CD8+ T-counts in the high PLR group and those in the low PLR group (18.9 ± 5.4 vs. 19.3 ± 3.3 *P* = 0.845; Figures 3B,D,G). While peritumoral stroma in the high PLR group has significantly more CD8^+^T-cells than that in the low PLR group (149.7 ± 40.3 vs. 83.5 ± 29.0 *P* = 0.001; Figures 3C,E,H). Potential associations of clinicopathological factors with CD8^+^T-cells counts in the peritumoral stroma were identified by logistic regression analysis. The median value of CD8^+^ T-cell counts in the peritumoral stroma was chosen as the cutoff point for distinguishing high CD8^+^ T-cell counts cases (*N* = 587) from low CD8^+^ T-cell counts cases (N = 587). After multivariate analysis, it was indicated that in addition to a high PLR level (≥150) (OR = 4.372; *P* < 0.001), a high Ishak inflammation score (≥3) (OR = 1.129; *P* < 0.001), cirrhosis (OR = 1.636; *P* = 0.001), and male (OR = 1.736; *P* = 0.009) was independently predictive of high CD8^+^ T-cell counts in the peritumoral stroma (Supplementary Table 7).

## Discussion

Many recent reports have found that a high PLR is relevant to an adverse prognosis in patients with different cancer types (5–7). In this study, a high PLR is related to more advanced and multiple malignant characteristics of HCC, higher HBV DNA value, more severe liver inflammation, more advanced tumor stage, and poorer clinical outcomes of HBV-related HCC after liver resection. The Kaplan-Meier analysis of RFS and OS in these HCC patients manifested that both tumor recurrence and survival rates are different significantly between the groups of patients with high and low PLR. Multivariate analysis showed that a high PLR worked as an independent and significant risk factor for recurrence and overall survival after curative liver surgery. More importantly, a high PLR showed good accuracy in predicting long-term survival in patients with early tumors. Further analysis indicated that PLR was the best predictor of recurrence, however, for OS, PLR combined with tumor diameter may be the best predictor.

BCLC Stage A is considered as an early stage of HCC, however, some of them still have poor survival outcome. Our findings indicate that stage A patients with a high PLR have a higher cumulative recurrence rate and a lower survival rate than those patients with a low PLR. Thus, the results of this study indicate that a high PLR is a factor predicting bad prognosis in patients with early stage HCC (Figures 1C,D). For the BCLC stage B patients, a high PLR substantially affects the prognosis of these patients after liver resection (Figures 1E,F).

Previous studies have shown that relatively depleted lymphocytes impair the host immune response to malignancy (21). Previous studies have proven platelets can interact with tumor cells and promote tumor growth (22, 23). Especially for HCC, platelets could produce vascular endothelial growth factor (VEGF) (24), platelet-derived growth factor (PDGF), (25) serotonin (26), and fibroblast growth factor (FGF) and its receptors which enhance HCC growth (27). Platelets can interact with a variety of different cell types, including endothelial and dendritic cells, T-lymphocytes, neutrophils, and mononuclear phagocytes. Interestingly, aspirin can inhibit platelet activation and thus reduce HCC development (28). In Asian countries, HBV infection is the primary risk for HCC development. Chronic HBV infection can cause persistent liver damage, fibrosis, and cirrhosis. The pathogenic mechanisms of HBV-related HCC not only involve viral factors, but also host factors as well (29). A functionally inefficient CD8^+^ T-cell response which causes a failure in virus clearance sustains a chronic necroinflammatory in liver and thus induces the carcinogenesis of HCC. During HBV infection, CD8^+^ T-cells are significant triggers of liver immunopathology and platelets play a significant role in immune-mediated liver injury by facilitating the accumulation of virus-specific CD8^+^ T-cells in the liver tissue. In this study, the results of immunohistochemical staining showed that the number of CD8^+^T-cells in the peritumoral stroma was significantly higher in the high PLR group than in the low PLR group. Multivariate logistic regression analysis indicated that a high PLR level (≥150), a high Ishak inflammation score, cirrhosis, and male patients was independently associated with a high CD8^+^ T-cell counts in the peritumoral stroma. These results suggest that a high PLR level, a high Ishak inflammation score, cirrhosis, and male patient are related to the level of liver inflammation. Interestingly, liver inflammation of male HCC patients was more severe than female patients, it is associated with the level of IL-6 (30).

In mice, researchers have found platelets that exacerbate hepatitis through serotonin secretion. This causes hepatic sinusoid microcirculation failure, which can lead to delayed viral clearance and CTL-mediated liver injuries (31). It was also found that platelet depletion can reduce CTL-mediated liver damage in mice (32). Platelet activation is necessary in such mechanisms because prostaglandin E1 treatment can reverse the effect of platelet reconstitution in platelet-depleted mice and thus weaken T cell-mediated liver injuries (33). Experiments have been performed verifying aspirin and clopidogrel, which is known also as platelet activation inhibitors, as mitigating necroinflammation and antigen-specific CTL accumulation in the liver of mice (34).

However, the mechanisms of recruiting platelets into the liver and thus facilitating the hepatic accumulation of CD8^+^ T-cells are still under covered. Research has found platelets adhere to sinusoidal hyaluronan via CD44 and circulate the CD8^+^ arrest by docking onto platelets. These CD8^+^ cells then propagate along liver sinusoids until hepatocellular antigens(AGS) are recognized (35). In addition, the activation-dependent expression of the platelet CD40 ligand contributes to the expansion phase of the virus-specific CD8^+^ T-cells, resulting in their accumulation at sites of infection (36). This suggests an direct interaction between activated platelets and CD8^+^ T-cells expressing CD40 (37, 38). Other authors have showed that the platelet CD40 ligand could potentially strengthen the virus-specific CD8^+^ T-cell responses through an indirect way, mostly by promotion of the maturation of dendritic cells (39). Our study demonstrated that high PLR is significantly correlated with a high aspartate aminotransferase level and high Ishak inflammation scores. Immunohistochemical analysis showed that a high PLR is significantly associated with high numbers of CD8^+^T-cells in the peritumoral stroma. Based on these observations, we reasoned that PLR reflects the severity of liver inflammation. These data provide direct evidence that PLR is an easily measurable inflammatory biomarker, and an elevated PLR is an independent predictor of survival outcome in HBV-related HCC subjects who have undertaken a liver resection.

The main limitation of this study is that this is a single institutional retrospective study, there may be potential biases in the collection of patients, so the results of this study need to be verified in further prospective studies, and relevant studies are in progress currently.

## Conclusions

Platelet-to-lymphocyte ratio is a practical and easily measurable prognosis marker for HBV–related HCC patients. Elevated PLR is significantly associated with the accumulation of CD8^+^ T-cells in peritumoral tissue. Our study indicates that PLR reflects the severity of liver inflammation.

## Data Availability Statement

All datasets generated for this study are included in the article/Supplementary Material.

## Ethics Statement

The studies involving human participants were reviewed and approved by Ethics Committee of the Eastern Hepatobiliary Surgery Hospital. The patients/participants provided their written informed consent to participate in this study. Written informed consent was obtained from the individual(s) for the publication of any potentially identifiable images or data included in this article.

## Author Contributions

YY and M-cW: data curation. TT and JH: formal analysis. W-pZ: funding acquisition and writing – original draft. S-xY, LL, PZ, and S-yF: investigation. F-mG, B-gJ, and F-cL: methodology. Z-yP: project administration. All authors contributed to the article and approved the submitted version.

## Conflict of Interest

The authors declare that the research was conducted in the absence of any commercial or financial relationships that could be construed as a potential conflict of interest.

## Abbreviations

HBV: hepatitis B virus
HCC: hepatocellular carcinoma
PLR: Platelet-Lymphocyte Ratio
SIR: systemic inflammatory response
CRP: c-reactive protein
NLR: neutrophil-to-lymphocyte ratio
LMR: lymphocyte to monocyte ratio
LT: liver transplantation
LR: liver resection
BCLC: Barcelona Clinic Liver Cancer
USG: ultrasonography
CT: computed tomography
MRI: magnetic resonance imaging
HBsAg: hepatitis B surface antigens
HBeAg: hepatitis B e antigens
HCV-Ab: hepatitis C virus antibody
AFP: alpha-Fetoprotein
CEA: carcinoembryonic antigen
CA19-9: carbohydrate antigen 19-9
ALT: alanine aminotransferases
AST: aspartate aminotransferases
PT: prothrombin time
TACE: transarterial chemoembolization
PRFA: percutaneous radiofrequence ablation
PEI: percutaneous ethanol injection
TMA: tissue microarray
PSM: Propensity score matching
RFS: recurrence-free survival
OS: overall survival
SD: standard deviation
ROC curve: receiver operating characteristic curve
HR: Hazard ratio
OR: Odds ratio
95% CI: 95% Confidence Interval
AGS: antigens.
