# Supplementary figures and images for: A High Preoperative Platelet-Lymphocyte Ratio Is a Negative Predictor of Survival After Liver Resection for Hepatitis B Virus-Related Hepatocellular Carcinoma: A Retrospective Study
Source: Front Oncol. 2020 Oct 16;10:576205. doi: 10.3389/fonc.2020.576205 (PMC7597590; doi:10.3389/fonc.2020.576205)

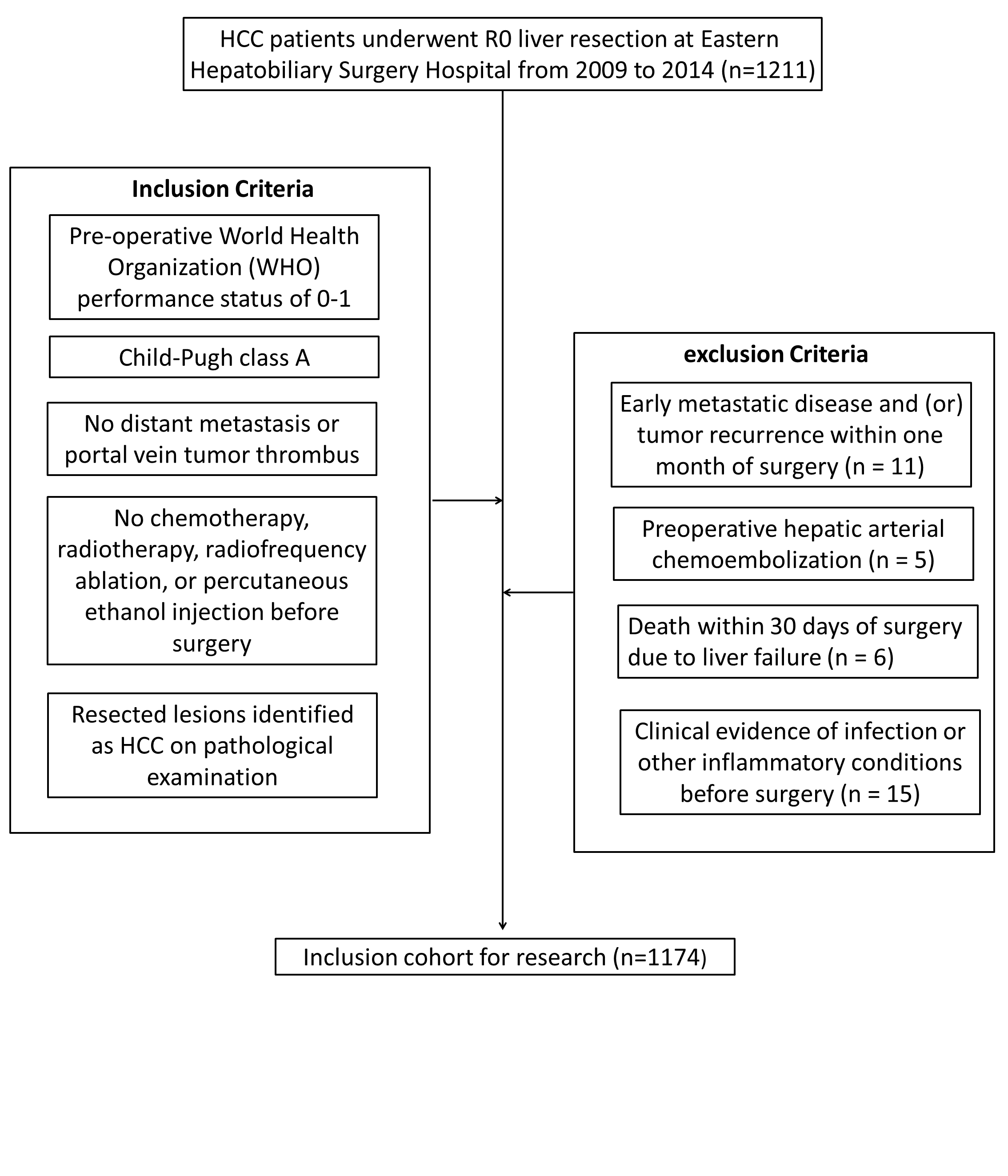

Supplement: Supplementary file 1 [file Image_1.TIF]

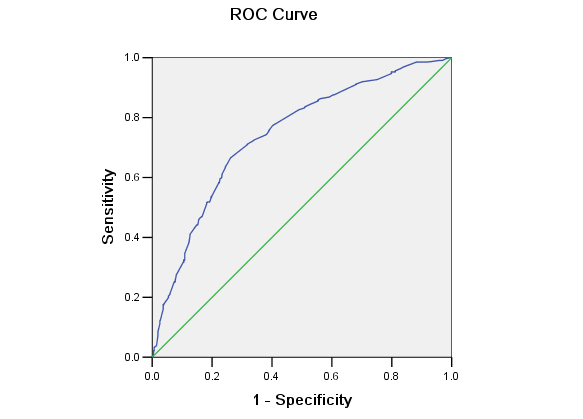

Supplement: Supplementary file 2 [file Image_2.TIF]

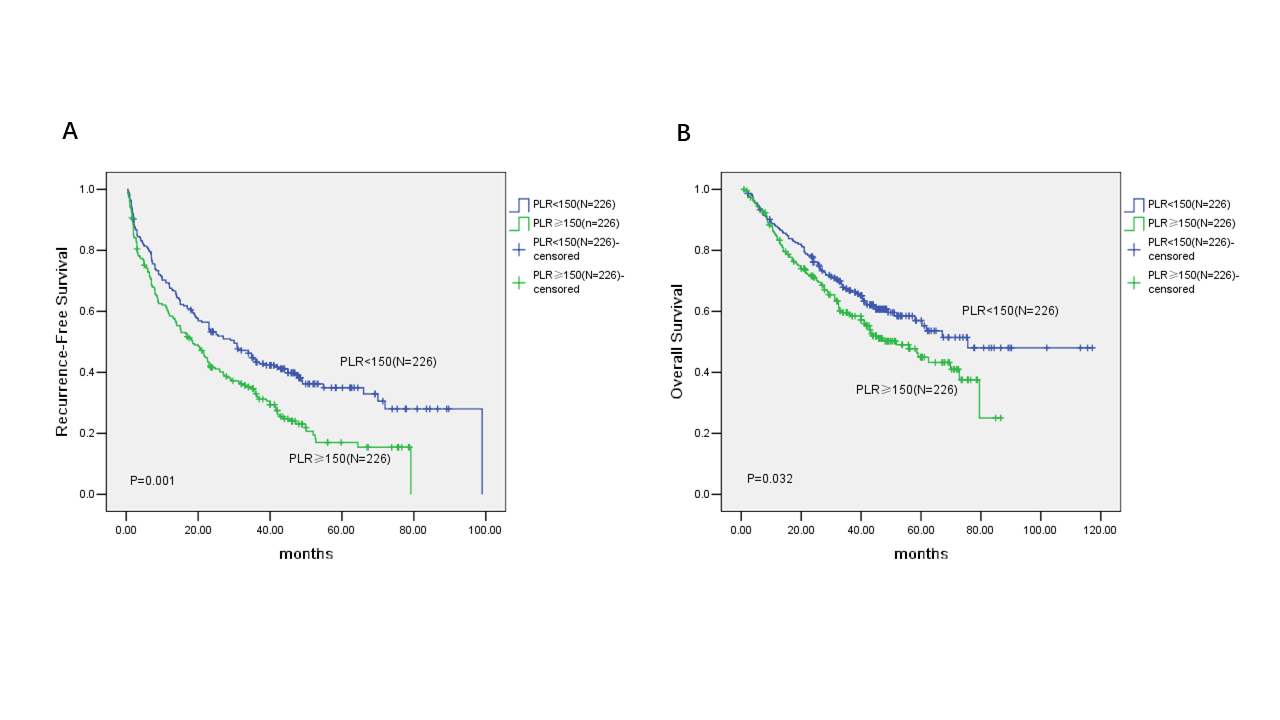

Supplement: Supplementary file 3 [file Image_3.TIF]
