# Supplementary material for: A High Preoperative Platelet-Lymphocyte Ratio Is a Negative Predictor of Survival After Liver Resection for Hepatitis B Virus-Related Hepatocellular Carcinoma: A Retrospective Study
Source: Front Oncol. 2020 Oct 16;10:576205. doi: 10.3389/fonc.2020.576205 (PMC7597590; doi:10.3389/fonc.2020.576205)
Supplement: Supplementary file 4 [file Data_Sheet_1.docx]

**Supplemental Figure Legends**

**Supplemental Figure. 1.** Selection of the study population.

**Supplemental Figure. 2** The ROC curve was performed to test the specificity and sensitivity of PLR in HCC patients. The area under the curve was 0.743. The sensitivity and specificity were 0.710 and 0.738, respectively.

**Supplemental Figure. 3** Survival curves of HCC patients with high PLR (≥150) and low PLR (<150) in the propensity score-matching (PSM) cohort. (A) The cumulative Recurrence-Free survival (RFS) curve of patients with high PLR (≥150) and low PLR (<150) in the propensity score-matching (PSM) cohort (P = 0.001). (B) The cumulative Overall Survival (OS) curve of HCC patients with high PLR (≥150) and patients with low PLR (<150) in the propensity score-matching (PSM) cohort (P = 0.032).

**Supplemental Table 1.Clinicopathological characteristics of the patients**

| **Characteristics** | **Total patients(N=1174)** |
| --- | --- |
| Gender |  |
| Male | 1035(88.16) |
| Female | 139(11.84) |
| Age(years)^a^ | 50(20-70) |
| Liver Cirrhosis |  |
| Yes | 713(60.73) |
| No | 461(39.27) |
| HBeAg |  |
| Positive | 281(23.93) |
| Negative | 893(76.07) |
| AFP(ng/ml) |  |
| ≥20 | 729(62.10) |
| <20 | 445(37.90) |
| Alanine aminotransferase(U/L) |  |
| ≥40 | 563(47.96) |
| <40 | 611(52.04) |
| Aspartate aminotransferase(U/L) |  |
| ≥40 | 521(44.38) |
| <40 | 653(55.62) |
| Total bilirubin(ummol/ml) |  |
| ≥17.1 | 423(36.03) |
| <17.1 | 751(63.97) |
| Albumin(g/L) |  |
| ≥35 | 1115(94.97) |
| <35 | 59(5.03) |
| HBV DNA(IU/ml) |  |
| ≥2000 | 552(47.02) |
| <2000 | 622(52.98) |
| Ishak inflammation score ^a^ | 6(2-14) |
| Ishak fibrosis score ^a^ | 4(1-6) |
| Tumor diameter(cm)^a^ | 4.9(0.5-22) |
| Tumor encapsulation |  |
| None | 649(55.28) |
| Complete | 525(44.72) |
| Major resection  Yes  No | 230(19.59)  944(80.41) |
| Microvascular invasion |  |
| Yes | 425(36.20) |
| No | 749(63.80) |
| Tumor number |  |
| Single | 653(55.62) |
| Multiple | 521(44.38) |
| Tumor differentiation |  |
| Ⅰ/II | 245(20.87) |
| III/Ⅳ | 929(79.13) |
| BCLC stage |  |
| 0+A | 695(59.20) |
| B | 479(40.80) |
| a Ages, Ishak inflammation score, Ishak fibrosis score, and tumor diameter are are shown as median (range). | |
| HBsAg, hepatitis B surface antigen; HBeAg, hepatitis B e antigen; AFP, alpha-fetoprotein; BCLC stage, Barcelona Clinic Liver Cancer stage. | |

**Supplemental Table 2. Comparisons of Clinicopathological and Demographic Characteristics of Patients With Elevated and Low PLR after propensity score matching (PSM)**

|  | Low PLR (<150), N=226 | Elevated PLR (≥150), N=226 | P-value |
| --- | --- | --- | --- |
| Gender |  |  |  |
| Male | 201(88.94) | 196(86.73) | 0.472 |
| Female | 25(11.06) | 30(13.27) |  |
| Age(years)^a^ | 50.44±10.59 | 49.83±10.93 | 0.547 |
| Liver Cirrhosis |  |  | 0.388 |
| Yes | 131(53.54) | 140(61.94) |  |
| No | 95(46.46) | 86(38.06) |  |
| HBeAg |  |  | 0.596 |
| Positive | 58(25.66) | 63(27.87) |  |
| Negative | 168(74.34) | 163(72.13) |  |
| AFP(ng/ml) |  |  | 1.000 |
| ≥20 | 152(67.26) | 152(67.26) |  |
| <20 | 74(32.74) | 74(32.74) |  |
| Alanine aminotransferase(U/L) |  |  | 0.851 |
| ≥40 | 118(52.21) | 116(51.33) |  |
| <40 | 108(47.79) | 110(48.67) |  |
| Aspartate aminotransferase(U/L) |  |  | 0.571 |
| ≥40 | 122(53.98) | 128(56.64) |  |
| <40 | 104(46.02) | 98(43.36) |  |
| Total bilirubin (ummol/ml) |  |  | 0.147 |
| ≥17.1 | 94(41.59) | 79(34.96) |  |
| <17.1 | 132(58.41) | 147(65.04) |  |
| Albumin(g/L) |  |  | 0.706 |
| ≥35 | 210(92.92) | 212(93.81) |  |
| <35 | 16(7.08) | 14(6.19) |  |
| HBV DNA(IU/ml) |  |  | 0.452 |
| ≥2000 | 115(50.88) | 123(54.42) |  |
| <2000 | 111(49.12) | 103(45.58) |  |
| Ishak inflammation score ^a^ | 4.93±2.62 | 5.25±2.68 | 0.334 |
| Ishak fibrosis score ^a^ | 4.61±2.99 | 4.16±2.72 | 0.178 |
| Tumor diameter(cm)^a^ | 8.22±4.48 | 8.31±4.11 | 0.808 |
| Tumor encapsulation |  |  | 0.847 |
| None | 138(61.06) | 136(60.18) |  |
| Complete | 88(38.94) | 90(39.82) |  |
| Major resection  Yes  No | 75(33.19)  151(66.81) | 68(30.09)  158(69.91) | 0.479 |
| Microvascular invasion |  |  | 0.566 |
| Yes | 89(39.38) | 95(42.04) |  |
| No | 137(60.62) | 131(57.96) |  |
| Tumor number |  |  | 0.345 |
| Single | 106(46.90) | 96(42.48) |  |
| Multiple | 120(53.10) | 130(57.52) |  |
| Tumor differentiation |  |  | 0.247 |
| Ⅰ/II | 23(10.18) | 31(13.72) |  |
| III/Ⅳ | 203(89.82) | 195(86.28) |  |

^a^ Age, Ishak inflammation and tumor diameter are expressed as mean±SD. PSM, propensity score matching;

HBeAg, hepatitis B e antigen; AFP, alpha-fetoprotein.

**Supplemental Table 3. Univariate and Multivariate analysis of factors associated with Recurrence-free survival of patients with HCC in the propensity score-matching (PSM) cohort.**

|  | Hazard ratio(95%CI) | P-value |
| --- | --- | --- |
| Univariate analysis |  |  |
| Gender(male vs female) | 0.805(0.577-1.124) | 0.202 |
| Age(Year)(≤60 vs >60) | 0.985(0.975-0.996) | 0.007 |
| Alanine aminotransferase(≥40U/L vs <40U/L) | 0.914(0.731-1.143) | 0.429 |
| Aspartate aminotransferase(≥40U/L vs <40U/L) | 1.280(1.020-1.606) | 0.033 |
| Albumin(<35g/L vs ≥35g/L) | 0.989(0.628-1.556) | 0.961 |
| HBV DNA(≥2000 IU /ml vs <2000 IU /ml) | 1.490(1.187-1.871) | 0.001 |
| Total bilirubin(≥17.1ummol/ml vs <17.1ummol/ml) | 1.056(0.840-1.328) | 0.640 |
| Ishak inflammation score(≥3 vs <3) | 1.345(1.019-1.774) | 0.036 |
| Ishak fibrosis score(≥3 vs <3) | 0.942(0.750-1.185) | 0.611 |
| PLR (≥150 vs <150) | 1.471(1.174-1.842) | 0.001 |
| AFP(≥20 vs <20 ng/ml ) | 1.643(1.280-2.109) | <0.001 |
| HBeAg(positive vs negative) | 1.315(1.021-1.694) | 0.034 |
| Tumor encapsulation(yes vs no) | 0.633(0.499-0.802) | <0.001 |
| Major resection(yes vs no) | 1.052(0.830-1.335) | 0.673 |
| Microvascular invasion(yes vs no) | 1.841(1.454-2.332) | <0.001 |
| Tumor number(multiple vs single) | 1.603(1.215-2.116) | 0.001 |
| Tumor differentiation(Ⅲ+Ⅳ vsⅠ+Ⅱ) | 1.456(0.993-2.135) | 0.054 |
| Tumor diameter(≥5cm vs <5cm) | 1.065(1.039-1.093) | <0.001 |
| Liver cirrhosis(yes vs no) | 1.322(1.056-1.655) | 0.015 |
|  |  |  |
| Multivariate analysis |  |  |
| Age(Year)(≤60 vs >60) | 0.993(0.983-1.004) | 0.228 |
| Aspartate aminotransferase(≥40U/L vs <40U/L) | 0.899(0.697-1.160) | 0.414 |
| HBV DNA(≥2000 IU/ml vs <2000 IU /ml) | 1.301(1.019-1.661) | 0.035 |
| Ishak inflammation score(≥3 vs <3) | 1.429(1.080–1.890) | 0.012 |
| PLR( ≥150 vs <150) | 1.487(1.182-1.870) | 0.001 |
| AFP( ≥20 vs <20 ng/ml ) | 1.243(0.949-1.628) | 0.115 |
| Tumor encapsulation(yes vs no) | 0.758(0.590-0.973) | 0.030 |
| Microvascular invasion(yes vs no) | 1.226(0.936-1.606) | 0.139 |
| Tumor number(multiple vs single) | 1.183(0.877-1.596) | 0.270 |
| Tumor diameter(≥5cm vs <5cm) | 1.054(1.023-1.085) | 0.001 |
| Tumor differentiation(Ⅲ+Ⅳ vsⅠ+Ⅱ) | 0.962(0.633-1.462) | 0.855 |
| Liver cirrhosis(yes vs no) | 1.318(1.021-1.700) | 0.034 |
| HRs(95% CI) and P values were calculated using univariate and multivariate Cox proportional hazard regression. | | |
| HBeAg, hepatitis B e antigen; AFP, alpha-fetoprotein; PSM, propensity score-matching | | |

**Supplemental Table 4. Univariate and Multivariate analysis of factors associated with Overall survival of patients with HCC in the propensity score-matching (PSM) cohort.**

|  | Hazard ratio(95%CI) | P-value |
| --- | --- | --- |
| Univariate analysis |  |  |
| Gender(male vs female) | 0.831(0.546-1.265) | 0.388 |
| Age(Year)(≤60 vs >60) | 0.991(0.977-1.004) | 0.172 |
| Alanine aminotransferase(≥40U/L vs <40U/L) | 0.998(0.754-1.322) | 0.991 |
| Aspartate aminotransferase(≥40U/L vs <40U/L) | 1.718(1.280-2.307) | <0.001 |
| Albumin(<35g/L vs ≥35g/L) | 0.803(0.481-1.338) | 0.399 |
| HBV DNA(≥2000 IU /ml vs <2000 IU /ml) | 1.635(1.225-2.184) | 0.001 |
| Total bilirubin(≥17.1ummol/ml vs <17.1ummol/ml) | 0.986(0.738-1.318) | 0.924 |
| Ishak inflammation score(≥3 vs <3) | 1.656(1.132-2.421) | 0.009 |
| Ishak fibrosis score(≥3 vs <3) | 0.794(0.596-1.057) | 0.114 |
| PLR (≥150 vs <150) | 1.345(1.015-1.782) | 0.039 |
| AFP(≥20 vs <20 ng/ml ) | 1.871(1.350-2.592) | <0.001 |
| HBeAg(positive vs negative) | 1.602(1.182-2.171) | 0.002 |
| Tumor encapsulation(yes vs no) | 0.609(0.449-0.826) | 0.001 |
| Major resection(yes vs no) | 1.094(0.852-1.406) | 0.481 |
| Microvascular invasion(yes vs no) | 1.778(1.328-2.381) | <0.001 |
| Tumor number(multiple vs single) | 2.001(1.444-2.773) | <0.001 |
| Tumor differentiation(Ⅲ+Ⅳ vsⅠ+Ⅱ) | 1.680(0.992-2.847) | 0.054 |
| Tumor diameter(≥5cm vs <5cm) | 1.096(1.062-1.130) | <0.001 |
| Liver cirrhosis(yes vs no) | 1.084(0.816-1.440) | 0.578 |
|  |  |  |
| Multivariate analysis |  |  |
| Aspartate aminotransferase(≥40U/L vs <40U/L) | 1.130(0.818-1.563) | 0.458 |
| HBV DNA(≥2000 IU/ml vs <2000 IU /ml) | 1.423(1.050-1.927) | 0.023 |
| Ishak inflammation score(≥3 vs <3) | 1.274(0.891-1.689) | 0.113 |
| PLR( ≥150 vs <150) | 1.309(1.201-1.738) | 0.041 |
| AFP( ≥20 vs <20 ng/ml ) | 1.345(0.948-1.909) | 0.097 |
| HBeAg(positive vs negative) | 1.285(0.940-1.757) | 0.116 |
| Tumor encapsulation(yes vs no) | 0.808(0.581-1.124) | 0.206 |
| Microvascular invasion(yes vs no) | 1.192(0.865-1.644) | 0.283 |
| Tumor number(multiple vs single) | 1.416(0.997-2.010) | 0.052 |
| Tumor diameter(≥5cm vs <5cm) | 1.067(1.031-1.106) | <0.001 |
| HRs(95% CI) and P values were calculated using univariate and multivariate Cox proportional hazard regression. | | |
| HBeAg, hepatitis B e antigen; AFP, alpha-fetoprotein; PSM, propensity score-matching | | |

| **Supplemental Table 5. The C-index of the predictors in Recurrence-free Survival and Overall Survival** | | | | | | | | | | | | | |
| --- | --- | --- | --- | --- | --- | --- | --- | --- | --- | --- | --- | --- | --- |
| Variables |  |  | RFS | | | |  | OS | | | | |  |
|  |  |  | C-index | 95%CI | P^†^-value | P^‡^-value |  | C-index | 95%CI | | P^†^-value | P^‡^- value |  |
| Nomogram | |  | 0.649 | 0.626-0.671 |  | <0.001 |  | 0.716 | 0.685-0.746 | |  | <0.001 |  |
| PLR |  |  | 0.549 | 0.534-0.564 | <0.001 |  |  | 0.559 | 0.538-0.579 | | <0.001 |  |  |
| AFP |  |  | 0.564 | 0.544-0.583 | <0.001 | 0.454 |  | 0.584 | 0.558-0.609 | | <0.001 | 0.018 |  |
| Tumor encapsulation | | | 0.561 | 0.542-0.580 | <0.001 | 0.076 |  | 0.591 | 0.565-0.618 | | <0.001 | <0.001 |  |
| Tumor diameter | |  | 0.582 | 0.563-0.602 | <0.001 | 0.599 |  | 0.635 | 0.609-0.661 | | <0.001 | <0.001 |  |
| HBV-DNA | |  | 0.542 | 0.523-0.562 | <0.001 | 0.064 |  | 0.569 | 0.543-0.595 | | <0.001 | 0.413 |  |
| Aspartate aminotransferase | | |  |  |  |  |  | 0.591 | 0.565-0.617 | | <0.001 | <0.001 |  |
| Tumor number | |  | 0.538 | 0.524-0.549 | <0.001 | 0.005 |  |  |  | |  |  |  |
| Ishak inflammation score | | | 0.567 | 0.549-0.586 | <0.001 | 0.505 |  |  |  |  |  |  |  |
| Microvascular invasion |  |  | 0.559 | 0.543-0.575 | <0.001 | 0.323 |  |  |  |  |  |  |  |
| AFP, alpha-fetoprotein |  |  |  |  |  |  |  |  |  |  |  |  |  |

P†-value: nomogram vs other predictors

P‡-value: PLR vs other predictor

| **Supplemental Table 6. The ROC of the predictors in Recurrence-free Survival and Overall Survival** | | | | | | | | | | | | | |
| --- | --- | --- | --- | --- | --- | --- | --- | --- | --- | --- | --- | --- | --- |
| Variables |  |  | RFS | | | |  | OS | | | | |  |
|  |  |  | AUC | 95%CI | P^†^-value | P^‡^-value |  | AUC | 95%CI | | P^†^-value | P^‡^-value |  |
| Nomogram | |  | 0.640 | 0.608-0.672 |  | 0.006 |  | 0.673 | 0.641-0.705 | |  | <0.001 |  |
| PLR |  |  | 0.561 | 0.539-0.583 | 0.004 |  |  | 0.559 | 0.533-0.585 | | <0.001 |  |  |
| AFP |  |  | 0.579 | 0.551-0.608 | <0.001 | 0.297 |  | 0.590 | 0.562-0.618 | | 0.003 | 0.105 |  |
| Tumor encapsulation | | | 0.566 | 0.537-0.595 | 0.001 | 0.787 |  | 0.599 | 0.570-0.629 | | 0.001 | 0.026 |  |
| Tumor diameter | |  | 0.568 | 0.539-0.597 | <0.001 | 0.649 |  | 0.624 | 0.595-0.653 | | 0.001 | <0.001 |  |
| HBV-DNA | |  | 0.567 | 0.538-0.596 | 0.032 | 0.723 |  | 0.584 | 0.555-0.613 | | <0.001 | 0.227 |  |
| Aspartate aminotransferase | | |  |  |  |  |  | 0.602 | 0.572-0.631 | | 0.001 | 0.029 |  |
| Tumor number | |  | 0.532 | 0.513-0.551 | <0.001 | 0.048 |  |  |  | |  |  |  |
| Ishak inflammation score | | | 0.563 | 0.536-0.591 | <0.001 | 0.879 |  |  |  | |  |  |  |
| Microvascular invasion |  |  | 0.571 | 0.547-0.595 | 0.019 | 0.547 |  |  |  | |  |  |  |
| **Combination** | |  |  |  |  |  |  |  |  |  |  |  |  |
| PLR |  |  | 0.561 | 0.539-0.583 | <0.001 |  |  | 0.559 | 0.533-0.585 | | <0.001 |  |  |
| PLR+AFP | |  | 0.612 | 0.582-0.641 | 0.056 | 0.030 |  | 0.618 | 0.587-0.648 | | 0.067 | 0.007 |  |
| PLR+Tumor encapsulation | | | 0.496 | 0.466-0.526 | <0.001 | 0.005 |  | 0.463 | 0.432-0.495 | | <0.001 | <0.001 |  |
| PLR+Tumor diameter | | | 0.593 | 0.563-0.622 | <0.001 | 0.006 |  | 0.637 | 0.606-0.668 | | 0.012 | <0.001 |  |
| PLR+HBV-DNA | |  | 0.603 | 0.574-0.632 | 0.019 | 0.001 |  | 0.616 | 0.586-0.646 | | 0.020 | 0.007 |  |
| PLR+ Aspartate aminotransferase | |  |  |  |  |  |  | 0.622 | 0.591-0.653 | | 0.049 | 0.007 |  |
| PLR+Tumor number | | | 0.574 | 0.548-0.599 | 0.021 | 0.128 |  |  |  |  |  |  |  |
| PLR+Ishak inflammation score | | | 0.597 | 0.568-0.627 | <0.001 | 0.001 |  |  |  |  |  |  |  |
| PLR+ Microvascular invasion | |  | 0.605 | 0.578-0.633 | 0.019 | 0.026 |  |  |  |  |  |  |  |

AFP, alpha-fetoprotein

P†-value: nomogram vs other predictors

P‡-value: PLR vs other predictor

**Supplemental Table 7. Logistic Regression Analysis of clinicopathological factors associated with a high PLR (N = 1174)**

|  | Odds ratio (95% CI) | P-value |
| --- | --- | --- |
| Univariate analysis |  |  |
| Gender(male vs female) | 1.610(1.121-2.309) | 0.010 |
| Age(Year)(≤60 vs >60) | 1.059(0.783-1.431) | 0.164 |
| PLR(≥150 vs <150) | 4.758(3.326-5.743) | <0.001 |
| Alanine aminotransferase(≥40U/L vs <40U/L) | 0.934(0.743-1.175) | 0.561 |
| Aspartate aminotransferase(≥40U/L vs <40U/L) | 1.434(1.138-1.807) | 0.002 |
| Albumin(<35g/L vs ≥35g/L) | 0.968(0.574-1.635) | 0.904 |
| HBV DNA(≥2000 IU /ml vs <2000 IU /ml) | 0.842(0.669-1.059) | 0.142 |
| Ishak inflammation score(≥3 vs <3) | 1.825(1.414-2.356) | <0.001 |
| Ishak fibrosis score(≥3 vs <3) | 1.152(1.110-1.196) | <0.001 |
| AFP(≥20 vs <20 ng/ml ) | 1.259(0.994-1.594) | 0.056 |
| HBeAg(positive vs negative) | 1.333(1.034-1.715) | 0.026 |
| Tumor encapsulation(yes vs no) | 0.611(0.485-0.769) | <0.001 |
| Major resection(yes vs no) | 1.164(0.832-1.516) | 0.271 |
| Microvascular invasion(yes vs no) | 1.303(1.001-1.696) | 0.049 |
| Tumor number(multiple vs single) | 1.134(0.813-1.582) | 0.459 |
| Tumor differentiation(Ⅲ+Ⅳ vsⅠ+Ⅱ) | 1.747(1.311-2.328) | <0.001 |
| Tumor diameter(≥5cm vs <5cm) | 2.589(2.046-3.275) | <0.001 |
| Liver cirrhosis(yes vs no) | 1.937(1.536-2.444) | <0.001 |
|  |  |  |
| Multivariate analysis |  |  |
| PLR(≥150 vs <150) | 4.372(3.946-5.812) | <0.001 |
| Ishak inflammation score(≥3 vs <3) | 1.129(1.057–1.207) | <0.001 |
| Liver cirrhosis(yes vs no) | 1.636(1.241-2.160) | 0.001 |
| Gender(male vs female) | 1.736(1.148-2.625) | 0.009 |
| Odds ratio(95% CI) and P values were calculated using univariate and multivariate Logistic Regression. | | |
| HBeAg, hepatitis B e antigen; AFP, alpha-fetoprotein | | |
